# 

**Published:** 1817-08

**Authors:** 


### CORRESPONDENCE.

JUVENIS is informed, in answer to his question, " what may be considered an *old* luxation of the humerus?" in reference to the propriety of attempting reduction in the individual case stated by him, that a period of " ten weeks" may fairly be considered to constitute an old luxation; nor is it probable that, under the circumstances related, " any violent efforts to reduce the bone into its proper situation" will be either safe or effectual. The unprincipled impostures of ignorant bone-setters are not peculiar to the part of the country where Juvenis resides. This genus is only to be extirpated when regular practitioners shall universally acquire the public confidence, by rendering themselves in every case more worthy of it.

Dr. Kennedy's description of an Intestinal Concretion is necessarily postponed, in consequence of the Editors not having yet received the promised account of its chemical analysis. They request that it may, if possible, be forwarded in time for insertion in the September number.

Dr. Palmer will shortly publish in this Journal, A Specimen of a New Method of Anatomical Arrangement, calculated, in his opinion, to facilitate the acquirement of an accurate and minute knowledge of Osteology—the basis of all anatomical science. This Specimen is taken from a system several years since framed by Dr. Palmer; and as his object in now publishing it is to ascertain the opinion of the public as to its practical utility and application, he respectfully solicits to it the attention of those who may not look with disdain on the labours of a country anatomist.

Mr. Stewart's Account of the late fatal Fever in the West Indies is received.

The Communications promised by Mr. Webster, Surgeon of the 51st Regiment, will be greatly esteemed.

Mr. Good's Nosological Work is under review; as is Magendie's Physiology.

Mayo's Remarks on Insanity are received, and will be reviewed in rotation.

☞ Authors and Publishers are requested to send Copies of new Works for Review as early as possible after their publication. They will be reviewed early in proportion.

